# Environment and Obesity in the National Children’s Study

**DOI:** 10.1289/ehp.11839

**Published:** 2008-09-12

**Authors:** Leonardo Trasande, Chris Cronk, Maureen Durkin, Marianne Weiss, Dale A. Schoeller, Elizabeth A. Gall, Jeanne B. Hewitt, Aaron L. Carrel, Philip J. Landrigan, Matthew W. Gillman

**Affiliations:** 1 Department of Community and Preventive Medicine; 2 Department of Pediatrics, Mount Sinai School of Medicine, New York, New York, USA; 3 Medical College of Wisconsin and Children’s Hospital of Wisconsin, Milwaukee, Wisconsin, USA; 4 Department of Population Health Sciences, University of Wisconsin, Madison, Wisconsin, USA; 5 College of Nursing, Marquette University, Milwaukee, Wisconsin, USA; 6 Interdepartmental Program in Nutritional Sciences, University of Wisconsin, Madison, Wisconsin, USA; 7 Marine and Freshwater Biomedical Sciences Center, University of Wisconsin-Milwaukee, Milwaukee, Wisconsin, USA; 8 Department of Pediatrics, University of Wisconsin, Madison, Wisconsin, USA; 9 Obesity Prevention Program, Department of Ambulatory Care and Prevention, Harvard Medical School and Harvard Pilgrim Health Care, Boston, Massachusetts, USA

**Keywords:** bisphenol A, built environment, endocrine disruptors, diet, National Children’s Study, obesity, phthalates, physical activity

## Abstract

**Objective:**

In this review we describe the approach taken by the National Children’s Study (NCS), a 21-year prospective study of 100,000 American children, to understanding the role of environmental factors in the development of obesity.

**Data sources and extraction:**

We review the literature with regard to the two core hypotheses in the NCS that relate to environmental origins of obesity and describe strategies that will be used to test each hypothesis.

**Data synthesis:**

Although it is clear that obesity in an individual results from an imbalance between energy intake and expenditure, control of the obesity epidemic will require understanding of factors in the modern built environment and chemical exposures that may have the capacity to disrupt the link between energy intake and expenditure. The NCS is the largest prospective birth cohort study ever undertaken in the United States that is explicitly designed to seek information on the environmental causes of pediatric disease.

**Conclusions:**

Through its embrace of the life-course approach to epidemiology, the NCS will be able to study the origins of obesity from preconception through late adolescence, including factors ranging from genetic inheritance to individual behaviors to the social, built, and natural environment and chemical exposures. It will have sufficient statistical power to examine interactions among these multiple influences, including gene–environment and gene–obesity interactions. A major secondary benefit will derive from the banking of specimens for future analysis.

Obesity is the consequence of a chronic net positive energy balance. The prevalence of obesity in American children has trebled in the past 30 years (Ogden et al. 2006; Strauss and Pollack 2001; Troiano et al. 1995). In 2003–2006, 31.9% of 2- to 19-year-olds had a body mass index (BMI) ≥ 85th percentile for age and sex (Ogden et al. 2008). This great increase in obesity portends future increases in incidence of heart disease (Bibbins-Domingo et al. 2007), diabetes (Lee et al. 2007), stroke, and possibly cancer (Bjorge et al. 2008) and is therefore projected to produce the first decline in U.S. life expectancy since the Great Depression (Olshansky et al. 2005). The recent explosive increase in prevalence of obesity reflects a complex interplay among *a*) changes in individual behaviors; *b*) changes in community structure, lifestyle, and the built environment; and *c*) possibly exposures to certain synthetic chemicals, such as endocrine disruptors (EDs), that may have the capacity to disrupt energy balance.

Control of the obesity epidemic will require understanding each of these factors and the interplay among them. This understanding will guide development of multi-pronged evidence-based strategies for obesity control. The goal of this review is to describe the approaches that the National Children’s Study (NCS) will employ to develop understanding of the causes of obesity, especially with regard to environmental factors.

## Background

Behavioral change is critical to the prevention and treatment of childhood obesity. Yet interventions against obesity that focus solely on modifying individual behavior to increase energy expenditure and/or reduce caloric intake in individual children have had limited success in sustaining weight loss or preventing obesity (Summerbell et al. 2005). A successful approach to reducing obesity and its comorbidities must also embrace understanding of community-level factors including the social, built, and natural environments. These environmental influences interact with a child’s diet, physical activity, genetic makeup, and metabolism (Meaney and Seckl 2004; Moll et al. 1991; Ong et al. 2007). An example of a multipronged approach that took careful cognizance of environmental influences is the success of the state of Arkansas in reducing obesity prevalence among school-age children. A thoughtful redesign of the school environment, with changes to school dietary options, implementation of universal physical education programs, and reduction of access to sugary soft drinks resulted in a decline in the prevalence of overweight children from 20.8% in the 2004–2005 school year to 20.4% in 2005–2006 (Anonymous 2007).

Access to safe play spaces may also influence activity patterns and thus reduce risk of obesity (Ewing et al. 2003; Frumkin et al 2004). Direct marketing to children (for example, through television ads during child-focused programming) encourages consumption of high-fat and high-sugar content foods and is a negative environmental influence (Gortmaker et al. 1996; Lobstein and Dibb 2005).

Unique windows of vulnerability have been identified for many of the environmental exposures linked to obesity (Ong et al. 2007). Fetal stressors such as maternal nutritional deprivation and smoking can result in intrauterine growth restriction (IUGR) and thereby influence hypothalamic–pituitary axis programming to increase future risk of obesity and diabetes (Meaney and Seckl 2004). Infants born to women with insulin-dependent diabetes are at higher risk of obesity, and milder, diet-controlled gestational diabetes may also increase risk (Bo et al. 2004; Dabelea et al. 2000). Maternal smoking during pregnancy is an independent risk factor for the development of childhood obesity (Bergmann et al. 2003; Oken et al. 2008). Excess gestational weight gain has been associated with increased child adiposity at 3 years of age in at least one prospective cohort (Oken et al. 2007). Exposure to endocrine-disrupting chemicals during pregnancy may enhance the risk for obesity in childhood (Newbold et al. 2007). Rapid weight gain during the first year of life (Reilly et al. 2005) and fewer hours of sleep during infancy (Taveras et al. 2008) further enhance the risk for the development of childhood obesity.

**Figure 1:**
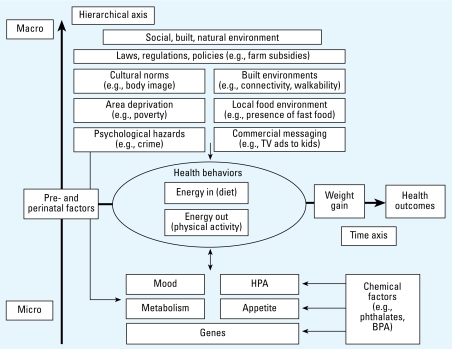
A life-course approach to childhood obesity. Abbreviations: BPA, bisphenol A; HPA, hypothalamic–pituitary axis. The life span is depicted horizontally, while factors are depicted at various levels hierarchically, from the individual-level factors in the lower part of the figure to the community-level factors in the upper part. Adapted from Glass and McAtee (2006).

Although previous cohort studies have contributed greatly to identifying many individual-level factors that contribute to the development of obesity in children and its persistence into adulthood both in the United States and in other countries (Berkey et al. 2000; Demerath et al. 2004; Freedman et al. 2005; Gordon-Larsen et al. 2006; Guo et al. 2002; Lake et al. 1997; Lauer et al. 1997; Moll et al. 1991; Nader et al. 2006; Nelson et al. 2006; Parsons 2001; Siervogel et al. 2000; Strauss and Knight 1999; Thompson et al. 2007), findings from those previous longitudinal studies have several limitations:

- Previous studies have not fully capitalized on the life-course approach to chronic disease epidemiology (Ben-Shlomo and Kuh 2002), an approach that embraces the concept that adult disease can have its origins in early life (or even fetal) exposures. Barker and Osmond (1986) promulgated this concept to account for an association between low birth weight and adult ischemic heart disease in Britain and Wales. The concept has been adopted increasingly in the epidemiologic approach to understanding chronic conditions (Lynch and Smith 2005) including obesity (Gillman 2004; James et al. 2006; Novak et al. 2006) and neurodegenerative conditions (Landrigan et al. 2005). The application of the life-course approach to identifying temporal relationships among risk factors for childhood obesity and their interaction is depicted in Figure 1. Multiple studies have documented unique windows of vulnerability to environmental hazards that may contribute to the causation of chronic conditions such as obesity (National Research Council 1993; Oken et al. 2008), yet few studies to date have collected the scope of data depicted in this figure at multiple points in the life span.
- Although the Centers for Children’s Environmental Health and Disease Prevention have collected data on environmental exposures to pregnant women and young children, these research centers have rarely focused on child weight status as an outcome (Wolff et al. 2008a). This weakness is especially relevant in light of new knowledge from animal studies, which suggest that endocrine-disrupting chemicals may modulate response to dietary intake (Bhathena and Velasquez 2002; Enan et al. 1996), disrupt the hypothalamic–pituitary axis (Rubin et al. 2001), and possibly increase risk for childhood obesity (Newbold et al. 2007).
- Although some studies have collected genetic data on participants and have been able to identify polymorphisms that increase risk for obesity, they have not simultaneously collected the data on environmental exposures that are necessary to examine carefully the interactions of genetic and environmental factors with diet and physical activity.
- Recent studies also suggest that obesity develops as a chronic condition much earlier than the school-age years (Kim et al. 2006). Earlier cohort studies that were first initiated when obesity in the preschool years was relatively infrequent are unlikely to provide data on exposures in early life that are essential to identify prenatal and early childhood risk factors for obesity.
- Many previous cohorts were limited in their capacity to identify risk factors for obesity that may be unique among Hispanics, a population for which obesity prevalence is increasing especially rapidly (Freedman et al. 2006; Strauss and Pollack 2001).
- Previous cohorts are limited in that they have not included sufficient numbers of children to draw contrasts between risk factors specific to rural and urban environments (Nelson et al. 2006).
- Past studies have been unable to allow accurate assessment of the role of access to parks and other places that encourage physical activity among children living in urban areas (Kipke et al. 2007).
- Many cohort studies were begun before the tripling of childhood obesity prevalence occurred (Kroke et al. 2006; Troiano et al. 1995; Wisemandle et al. 2000)—a trend increasingly attributed to the collective effect of community-level factors for which policy changes may be the only effective means for preventing further increases in obesity prevalence (Summerbell et al. 2005). To assess the impact of these more recent community-level factors, new cohorts in which these risk factors exist are needed.
- Although studies from other countries, such as the Avon Longitudinal Study of Parents and Children (Moll et al. 1991; Ong et al. 2007) and the Danish National Birth Cohort (Olsen et al. 2001), will provide important insights into the etiology of childhood obesity, the environmental factors that contribute to obesity in American children are likely to be different, and the pool of genetic polymorphisms that modify risk may be much different from that of European children.

## Progress of the NCS

**Figure 2:**
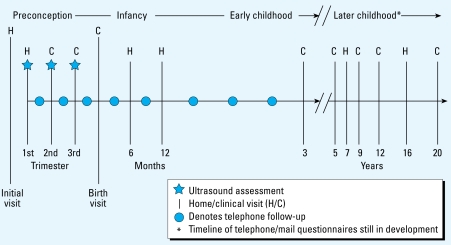
Schedule of visits, NCS. Stars denote ultrasound assessment, while | on the timeline represents home/clinical assessments (denoted by H/C). Circles denote telephone follow-ups, and asterisk denotes components of the timeline for telephone and mail questionnaires that are still under development.

In response to increases in the prevalence of obesity and a number of other chronic conditions, the U.S. Congress, through the Children’s Health Act of 2000, authorized the National Institute of Child Health and Human Development (NICHD) “to conduct a national longitudinal study of environmental influences (including physical, chemical, biological, and psychosocial) on children’s health and development” (Children’s Health Act 2000). The design of the NCS has been extensively described elsewhere (Branum et al. 2003; Landrigan et al. 2006; Trasande and Landrigan 2004; Trasande et al. 2006). With assistance from the staff of the National Center for Health Statistics at the Centers for Disease Control and Prevention, NCS staff developed a multistage clustered sampling approach to enroll a sample of 100,000 live births representative of all American children (Strauss et al. 2004). Families who are enrolled in the study will participate in a minimum of 13 data collection encounters: at least one visit before conception; two times during pregnancy; at birth; at 6, 12, and 18 months of age in early childhood; at 3, 5, 7, 9, and 12 years of age in childhood; and at 16 and 20 years of age in adolescence (Figure 2). Figure 2 depicts the timeline of visits across the complete study, and Tables 1 and 2 describe the measurements planned for preconception through 3 years of age for the seven Vanguard (pilot) locations. Enrollment of women will occur in 105 primary sampling units (counties or, in the case of more sparsely populated areas, clusters of counties) and began in January 2009.

The mission of the NCS is to provide the federal government with a scientifically robust guide to disease prevention, and to assure scientific rigor the study has always been hypothesis-driven. The topical working groups convened by the NCS Advisory Committee developed initial core hypotheses for the study, in consultation with thousands of scientists and representatives from community groups and professional organizations. A current list of hypotheses with supporting scientific rationales that were accepted and refined by the Interagency Coordinating Committee [composed of senior scientists from NICHD, the National Institute of Environmental Health Sciences, the Centers for Disease Control and Prevention, and the U.S. Environmental Protection Agency (EPA)] is available on the NCS website (NCS 2008).

Childhood obesity is a lead focus of the NCS and is addressed in 6 of 30 core hypotheses. Table 3 presents the gaps of knowledge that remain with respect to four of these core hypotheses: obesity and insulin resistance from impaired maternal glucose metabolism; obesity and insulin resistance associated with IUGR; breast-feeding associated with lower rates of obesity and lower risk of insulin resistance and fiber; and whole grains, high glycemic index, insulin resistance, and obesity.

Table 3 also presents how the NCS will address these gaps through its design. In this review, we highlight how the study will provide important new knowledge with regard to two core hypotheses that link factors in the chemical and built environments with childhood obesity.

## Obesity-Related Hypotheses of the NCS

### Impact of neighborhood environment on risk of obesity and insulin resistance

Built environment features such as mixed land use, increased proximity to recreational activities and green space, as well as safety (e.g., low crime rates and perceived traffic safety for pedestrian and bicyclists) have been associated in cross-sectional studies with increased physical activity (Cervero and Duncan 2003; Ellaway et al. 2005; Li et al. 2005) and lower risk of obesity among adults (Ewing et al. 2006; Frank et al. 2004; Lopez 2004). Few studies have examined the impact of the built environment on younger children, and those studies have focused upon circumscribed geographic areas and/or socioeconomically advantaged and ethnically homogeneous communities (Papas et al. 2007). Decreased access to healthy eating choices in low socioeconomic status neighborhoods has been documented in at least two studies (Galvez et al. 2008; Morland et al. 2006). Factors such as climate and topography have been taken into account infrequently (Timperio et al. 2006). The effect of after-school and summer adult-organized programs on obesity and insulin resistance is unknown. In the absence of such programs, parents living in urban areas may instruct their children to go directly home from school where indoor activities are largely limited to watching television and playing computer games in the security of the home.

A systematic review of previous studies of the built environment and childhood obesity identified inconsistencies in measurements of the built environment across studies and cross-sectional designs as major deficits of previous studies, and noted that these studies rarely studied both diet and physical activity (Papas et al. 2007). Because of its focus on community characterization (Landrigan et al. 2006), the NCS will allow more careful identification of those features of neighborhoods that affect physical activity and diet, such as proximity to play spaces, availability of healthy food stores, and neighborhood walkability.

The NCS represents a major opportunity to explore the role of specific aspects of the neighborhood environment at different periods in a child’s development. Access to safe play spaces near a child’s home, for example, may be especially protective against obesity during the early school years, but less so during adolescence. The design of the NCS capitalizes on the life-course approach and allows for separate analyses of the impact of certain factors on the development of obesity or increase in adiposity within certain time periods. Simultaneous collection of socioeconomic and genetic data as well as measures of diet and physical activity (Tables 1 and 2) will permit careful distinction of the role of certain environmental risk factors during each window of vulnerability.

### Chemical environmental agents and the endocrine system

The impact of EDs on humans was first identified by Herbst and Bern, who observed eight cases of clear cell adenocarcinoma of the vagina in young women who had been exposed *in utero* to diethylstilbestrol (DES), a synthetic estrogen prescribed to pregnant women in the 1950s, 1960s, and 1970s to prevent miscarriage (Bern 1992). Prenatal exposure to DES has been found subsequently to induce obesity in an animal model (Newbold et al. 2007). Identification of endocrine-disrupting chemicals has been limited by the lack of toxicity testing data available for many chemicals in widespread use (U.S. EPA 1998).

Because so few chemicals have been tested for their toxicity, the possibility exists that other chemicals besides DES influence somatic growth and obesity (Bhathena and Velasquez 2002; Rubin et al. 2001). One potential endocrine-disrupting chemical, bisphenol A (BPA), is used to manufacture polycarbonate resin in the coatings of food and beverage containers (Brotons 1995). Exposure to BPA, phthalates, and other EDs is widespread in American children (Centers for Disease Control and Prevention 2005), and animal studies increasingly suggest the potential for toxicity at current levels of exposure (Vom Saal and Hughes 2005). *In vitro* studies have found that BPA induces fibroblast differentiation into adipocytes (Masuno et al. 2002). Animal studies have found that BPA affects glucose transport in fat cells (Sakurai et al. 2004). BPA also disrupts glucagon secretion in intact Langerhans cells at nanomolar levels (Alonso-Magdalena et al. 2005). These studies raise the possibility that BPA could be a risk factor for the development of obesity, a question undergoing examination in at least one Center for Children’s Environmental Health and Disease Prevention (Wolff et al. 2008).

Phthalates are used in a variety of personal care products such as shampoos and in the synthesis of polyvinyl chloride (Sathyanarayana 2008). Phthalates have been documented consistently in animal studies to have antiandrogenic effects (Bell 1982; Fisher 2004; Parks et al. 2000). Cohort studies have begun to assess for potential effects in humans and suggest susceptibility at lower levels of exposure than those documented to have effects in animals. It is hypothesized that the most severe effects may be associated with exposures in prenatal and early postnatal life. Decreases in anogenital distance among infant males have been associated with elevated urinary phthalate levels during pregnancy (Swan et al. 2005), and breast milk levels of monoester phthalates have been associated with higher serum hormone binding globulin levels and luteinizing hormone to free testosterone ratios (Main et al. 2006). Diminished sperm motility has been identified among exposed men (Duty et al. 2003; Hauser 2006; Hauser et al. 2006), and low-molecular-weight phthalates have been associated with increased birth weight and longer duration of gestation in at least one birth cohort (Wolff et al. 2008b). Although few studies have analyzed the impact of phthalate exposure on increased adiposity in children, analysis of the 1999–2002 National Health and Nutrition Examination Survey has identified increases in urinary phthalate levels among men with increased waist circumference and homeostatic model assessment, a measure of insulin resistance (Stahlhut et al. 2007).

Lack of accurate information on the level and timing of past exposures to EDs has been the principal limitation of most previous studies of the potential human impacts of EDs. This limitation will be directly addressed by the prospective design of the NCS. In the NCS, exposures to chemicals will be measured during pregnancy, in breast milk, and in the perinatal period before the appearance of health effects. The large sample size will facilitate investigation of possible links between low-prevalence endocrine-disruptor exposures and health outcomes, and state-of-the-art laboratory assessment of chemical exposures will further sharpen the study's ability to discern effects of exposures to EDs. The large sample size will also permit study of genetic polymorphisms and gene–environment interactions, which may unearth individual differences in susceptibility to EDs. As new EDs are identified, specimens can be withdrawn from the NCS repository to analyze their content for appropriate biomarkers to assess whether these EDs may be risk factors in the development of obesity (Landrigan et al. 2003).

## Conclusion

The NCS presents previously unrealized opportunities for the identification of risk factors for childhood obesity, and for their subsequent elimination through prevention. Just as the Framingham Heart Study provided health care providers with hitherto novel information on risk factors for cardiovascular disease that enabled them to offer evidence-based advice to limit smoking, reduce the intake of fatty foods, and control hypertension, the NCS will suggest interventions that can be used to prevent obesity by communities, policy makers, and child health providers. A major strength of the study is that it will be representative of American children. It is anticipated, for example, that > 20,000 children in the cohort will be Hispanic, permitting examination of unique risk factors among a subgroup that has been disproportionately affected by the epidemic.

The hypotheses presented in this review cover only a small percentage of the findings likely to emerge from the NCS. The core NCS hypotheses are dynamic, and as the study is implemented, new questions will emerge and result in modifications to the study protocol. Others may be clearly answered through the NCS or other studies, or become outdated as the whole body of knowledge adjusts the direction of inquiry. For some areas of inquiry where the science is in relatively nascent stages, the major benefits to be gained from the study derive from its hypothesis-generating nature. The NCS will provide a major opportunity to confirm putative genetic links identified in other studies through the study of genetic sequences of children and their families (Landrigan et al. 2008). As new putative EDs are identified, subsamples of biospecimens stored at the NCS Specimen Repository can be rapidly analyzed to test for associations in a large-scale cohort that represents the population of U.S. children.

Of course, no observational study by itself can demonstrate causality. The NCS will identify risk factors for which causality may be suggested on the basis of strength, consistency, temporality, biological gradient, and plausibility. Findings from the NCS will prompt further interventions such as randomized controlled trials, policy interventions, and other initiatives that will confirm or refute the role of identified risk factors in the development of obesity and its associated comorbidities.

The life-course approach underlying the design of the NCS may very well lead to delineating the duration and impact of environmental, behavioral, and social exposures on risk for obesity. No study will have followed women from preconception and subsequently followed their children at such frequent intervals early in childhood and then through adolescence and young adulthood. The NCS will collect an array of biospecimens, dietary and physical activity data, and social and chemical environmental factors on all 100,000 children for all proposed data collection time points, whereas other cohorts have collected more limited data at each time point or collected complete data on a smaller sample.

A major challenge of the NCS will be to overcome the difficulties in measuring physical activity, diet, and anthropometry in children that have bedeviled past studies. Limitations of reliability and validity do exist with food-frequency questionnaires (Coates and Monteilh 1997; Teufel 1997) and other instruments commonly used to measure dietary intake, although promising alternatives have been developed for populations in which past instruments have not proven reliable (Yaroch et al. 2000). The vagaries of collecting information on physical activity by questionnaire are well documented (Kohl et al. 2000), but accelerometry and other measuring techniques are increasingly promising in their precision and application (Ekelund et al. 2001; Janz et al. 2002). BMI is not a perfect measure of adiposity (Pietrobelli et al. 1998), and dual-absorption X-ray absorptiometry has been strongly correlated with cardiovascular disease factors in children (Lindsay et al. 2001). Bioimpedance analysis and skinfold thickness are increasingly used to measure adiposity (Gutin et al. 1996; Kettaneh et al. 2005).

These challenges will not be easily dismissed, and the opportunity is ripe for contributions from the obesity research community to ensure that the best questionnaires and measurement approaches are utilized in an efficient and cost-effective way. At this time, the protocol has been finalized only for the seven Vanguard (pilot) locations, and even for those locations only through birth. The NCS also offers major opportunities to study the validity and reliability of alterative measurement approaches through adjunct studies in collaboration with existing study centers. These studies may use the full or a subsample of the study cohort, with the caveat that proposed new data collection not impose undue additional burden on study participants or additional financial burden on the study.

The NCS will also trigger ancillary and follow-up studies and provide the next generation of obesity researchers opportunities to apply for funding (Lyman et al. 2005). The NCS will make public use, deidentified data sets available in accordance with federal privacy regulations.

Previous cohort studies of cardiovascular risk have plowed the terrain to identify major risk factors and allow the NCS to close in on solutions to the epidemic of childhood obesity. However, they have also demonstrated that these relationships are complex and temporally dependent, making a large longitudinal cohort study beginning in the prenatal period essential. The NCS thus offers us great hope in combating the obesity epidemic among America’s children.

## Figures and Tables

**Table 1:** NCS proposed measurements from preconception through pregnancy.

	Preconception					Pregnancy							
Measurement	Initial	Initial follow-up	First month	Second month	Fourth month	First trimester (< 14 weeks)	First trimester follow-up (< 14 weeks)	First trimester ultrasound	16–17 weeks	Second trimester (22–24 weeks)	Third trimester (28–32 weeks)	Third trimester follow-up	36 weeks
Location/type	Home	Mail	Phone	Phone	Phone	Home	Mail	Clinic	Phone	Clinic	Clinic	Mail back	Phone
Body composition													
Length/height													
Weight	M					M, F				M	M		
Head circumference													
Arm circumference	M					M, F				M	M		
Waist circumference	M					M, F				M	M		
Hip circumference	M					M, F				M	M		
Leg length													
Skin folds	M					M, F				M	M		
Ultrasound						M^a^		M^a^		M	M		
Blood pressure	M					M, F				M	M		
Bioimpedance analysis													
Diet													
Community-based food collection						M, N							
Food frequency questionnaire							M					M	
Self-completion diary	M	M	M	M	M	M	M	M	M	M	M	M	M
Activity measures													
Activity questionnaire													
TV viewing													
Time outdoors													
Activity diary													
Biological specimens													
Vaginal swabs	M					M					M		
Blood	M					M, F					M		
Urine (self-collected)		M					M, F				M		
Saliva (self-collected)							M					M	
Hair	M					M, F					M		
Cord blood													
Umbilical cord and placenta													
Meconium													
Breast milk													
Socioeconomic/environmental data													
Mother/father education/ SES/housing	M	M	M	M	M	M, F	M	M	M	M	M	M	M
Medical provider visit log	M	M	M	M	M	M	M	M	M	M	M	M	M
Medical record/chart abstraction													

Abbreviations: F, data from father; M, data from mother; N, neighborhood level data; SES, socioeconomic status.

a Data to be abstracted from clinical ultrasound if available; otherwise ultrasound to be performed on mother in clinic setting as part of NCS.

**Table 2:** NCS proposed measurements from birth through 3 years of age.

	Birth	Neonate	Childhood									
	Delivery	Predischarge visit	3 months	6 months	6-month follow-up	9 months	12 months	12-month follow-up	18 months	24 months	30 months	36 months
Location/type	Hospital	Hospital	Phone	Home	Mail back	Phone	Home	Mail back	Phone	Phone	Phone	Clinic
Body composition												
Length/height		C		C			C					C
Weight		C		C			C					C
Head circumference		C		C			C					C
Arm circumference		C		C			C					C
Waist circumference		C		C			C					C
Hip circumference		C		C			C					C
Leg length												C
Skin folds		C		C			C					C
Ultrasound												
Blood pressure							C					C
Bioimpedance analysis												C
Diet												
Community-based food collection									C, N			C, N
Food frequency questionnaire				M				M				C
Self-completion diary												C
Activity measures												
Activity questionnaire												C
TV viewing												C
Time outdoors												C
Activity diary												C
Biological specimens												
Vaginal swabs												
Blood	M, C						C					C
Urine (self-collected)	M			C			C					C
Saliva (self-collected)					M, F			C				C
Hair				C			C					C
Cord blood	C											
Umbilical cord and placenta	M											
Meconium		C										
Breast milk			M		M				M			C
Socioeconomic/environmental data												
Mother/father education/SES/housing			M	M, F	M	F	M	M	F	M	M	M, F
Medical provider visit log			M	M	M	M	M	M	M	M	M	C
Medical record/chart abstraction		M, C										C

Abbreviations: C, data from child; F, data from father; M, data from mother; N, neighborhood level data; SES, socioeconomic status.

**Table 3:** Core hypotheses of the National Children’s Study relating to obesity.

Hypothesis domain	Obesity and insulin resistance from impaired maternal glucose metabolism	Obesity and insulin resistance associated with intrauterine growth restriction	Breast-feeding associated with lower rates of obesity and lower risk of insulin resistance	Fiber, whole grains, high glycemic index and obesity, insulin resistance
Relevance	If gestational diabetes (or excessive gestational weight gain) is conclusively demonstrated to increase risk of childhood obesity/insulin resistance, then prevention of overweight among women of childbearing age may be especially useful in the prevention of childhood obesity.	If IUGR is identified as a preventable cause of obesity, then prevention of IUGR could form a major component of obesity prevention in the United States.	In the absence of proven alternatives, breast-feeding could serve as a lead component of obesity prevention in the United States. Because breast-feeding initiation, exclusivity, and continuation vary greatly by race and ethnic group, breast-feeding could also be a major causative factor for existing and widening disparities in prevalence of childhood obesity and its comorbidities, and targeted interventions among populations where breast-feeding is less frequent would be urgently indicated.	The role of glycemic content in modulating response to an energy load is of tremendous interest in the policy community. Soft drink consumption by children is on the rise, and easy access in some schools is cited as a possible exacerbating factor to the obesity epidemic. The most recent USDA Dietary Guidelines now encourages three ounces/day whole grain intake, but this amount of intake may not be sufficient to reduce risk.
Gaps in state of knowledge	Most studies have had small sizes, and have not completely differentiated severe, insulin dependent and mild diet-controlled gestational diabetes. Follow-up has typically been limited to the offspring preschool years, thus precluding documentation of longer term effects on child body composition and metabolic status.	Most studies of IUGR and adult insulin resistance are based on historical data, and limited to information about size at birth and adult outcomes, with no information available about different periods during prenatal development. Results have been contradictory because of differing definitions of key dependent and independent variables, use of different measurements, and limitation on the period of follow up. Many apparent confounders for this phenomenon (e.g., levels of such hormones as cortisol and insulin-like growth factors) are likely embedded in the same causal framework with IUGR that underlies the fetal origins of later life phenomena. Few studies have serially measured fetal size and growth using ultrasound.	If breast-feeding is protective for childhood obesity, it is unclear whether this is due to constituents of breast milk, metabolic programming, regulation/control of intake by mother and/or infant, or aspects of family lifestyle/home environment that are different for breast- and formula-fed infants. Measurement of family-level confounders appears to be extremely important, and has been lacking in previous studies of breast-feeding and obesity. Studies do suggest that breast-feeding may only proffer protection from future risk of obesity in certain subpopulations.	Studies of the role of glycemic index to date have been limited to small samples, and because the duration of follow-up has typically been brief, the applicability of these findings to broad populations of children has been limited. The contribution of sugary snacks and drinks to current prevalence is unknown, and studies to date have not had the statistical power to isolate for confounding with caloric intake, genetics, physical activity among other factors, or to examine the possibility of specific windows of vulnerability with regard to high glycemic content. Few studies have assessed the impact of whole grains on risk of obesity and insulin resistance in younger children.
Unique capacity of the National Children’s Study	A cohort of 100,000 is adequate for assessment of main effects for exposures at least as prevalent as maternal gestational diabetes, and outcomes at least as prevalent as adolescent type 2 diabetes. It is certainly not too large, as power becomes marginal for main effects within sex and race/ethnicity-specific strata, when exposures are as uncommon as gestational diabetes, even for relatively common outcomes such as obesity, for odds ratios < 1.5.	The National Children’s Study design will measure maternal nutritional status and fetal stressors at different periods during prenatal development; fetal growth measured with serial ultrasounds; fetal body composition; size and body composition at birth and throughout childhood, adolescence and early adulthood; dietary intake of mother during pregnancy and the offspring postnatally; and key hormonal levels in the mother and child. Information about family factors (e.g., sibling birth size, body composition of other family members, maternal history of birth size) will better control confounding.	Prospective report of breast-feeding, and use of a metric that incorporates duration of breast-feeding with the percentage of intake derived from breast milk will settle existing debates about the protective benefit offered by breast-feeding. Collection of genetic data will provide an opportunity to identify whether genetic or other factors influence the relationship between breast-feeding and obesity/insulin resistance among whites and nonwhites. The NCS will follow a large multiethnic population and have the power to assess the influence of cultural factors on breast-feeding and formula supplementation.	The National Children’s Study offers strong statistical power to examine the role of factors in the dietary environment of children, and is the first large cohort study with the potential to use the knowledge produced by the Human Genome Project to examine the role of genetic vulnerability in modifying the risk posed by factors such as glycemic index.
